# A New Method to Sort Differentiating Osteoclasts into Defined Homogeneous Subgroups

**DOI:** 10.3390/cells11243973

**Published:** 2022-12-08

**Authors:** Philippa A. Hulley, Helen J. Knowles

**Affiliations:** Botnar Institute for Musculoskeletal Sciences, Nuffield Department of Orthopaedics Rheumatology & Musculoskeletal Sciences, University of Oxford, Oxford OX3 7LD, UK

**Keywords:** osteoclast, live cell sorting, bone resorption, osteoclastogenesis, collagen gel

## Abstract

Osteoclasts regulate skeletal development but also drive pathological osteolysis, making them prime therapeutic targets. Osteoclast research is limited by the heterogeneity of osteoclast populations generated in vitro, where the mixture of undifferentiated monocytes, binuclear pre-osteoclasts and multinucleated osteoclasts has by necessity been considered a single osteoclast population. This study describes the differentiation of primary human CD14+ monocyte-derived osteoclasts in 3D collagen gels. These osteoclasts remained small (>95% with ≤5 nuclei) but were viable and active; when released from the gel with collagenase, they fused rapidly when reseeded onto solid substrates and resorbed dentine for 2–3 weeks. 3D-generated osteoclasts expressed cell surface markers of osteoclast differentiation (e.g., CD9, RANK, OSCAR, CD63, CD51/61) which, with their small size, enabled live cell sorting of highly enriched viable subpopulations of human osteoclasts that retained full functional resorption capacity. Low-yield osteoclast preparations were strongly enriched to remove undifferentiated cells (e.g., 13.3% CD51/61+ to 84.2% CD51/61+), and subpopulations of CD9+CD51/61− early osteoclasts and CD9+CD51/61+ mature cells were distinguished. This novel approach allows the study of selected populations of differentiating osteoclasts in vitro and opens the door to in-depth transcriptomic and proteomic analysis of these cells, increasing our ability to study human osteoclast molecular mechanisms relevant to development, aging and disease.

## 1. Introduction

Osteoclasts are large multinucleated cells that resorb bone and play an essential role in skeletal development and maintenance. However, their over-activation drives pathological osteolysis in conditions including osteoporosis, cancer, rheumatoid arthritis and other chronic inflammatory conditions, making them a prime target for therapeutic intervention [1]. The limitations of current anti-resorptive strategies, particularly in oncology and chronic inflammatory disease, drive the need to develop novel osteoclast-targeting agents [2,3]. However, the necessary use of powerful and sensitive new technologies for in-depth sequencing and proteomic interrogation of osteoclasts is hampered by the technical failure of current methods to deliver pure populations of viable cells.

Osteoclasts cannot be directly isolated ex vivo due to their tight adhesion to bone, and their rarity, except in chickens, rabbits [4] and human pathological conditions such as giant cell tumour of bone [5]. Osteoclast studies are therefore performed in vitro; precursor cells are most often obtained from the mixed cell populations of murine bone marrow, or the peripheral blood mononuclear cell (PBMC) fraction of blood from human donors.

Osteoclasts form by the fusion of CD14+ monocyte or macrophage precursors [6,7], in the presence of macrophage colony stimulating factor (M-CSF) and receptor activator of nuclear factor kappa B ligand (RANKL), to produce mature multi-nucleated cells [8,9,10]. M-CSF induces RANK expression on the surface of precursor cells. This binds to RANKL, leading to the early activation of nuclear factor of activated T-cells, cytoplasmic 1 (NFATc1), a transcription factor which, in turn, sequentially transcribes osteoclast-specific genes such as tartrate-resistant acid phosphatase (TRAP), pro-fusion DC-STAMP, CD51/61 (αvβ3 integrin, vitronectin receptor), calcitonin receptor and cathepsin K [11]. Sequential fusion is another characteristic of osteoclastogenesis that can be separated from the induction of osteoclast-specific genes. Initial fusion occurs between two mononuclear cells; however, the most common fusion event is between the resulting multinucleated cells and other mononuclear cells. Indeed, different molecular mechanisms of fusion generate small binuclear pre-osteoclasts and mature (≥3 nuclei) osteoclasts; CD47 promotes the fusion of mononuclear monocytes, whereas syncytin-1 stimulates the generation of larger osteoclasts via fusion between mononuclear and multinucleated cells [12].

The cell population produced by in vitro osteoclast culture methods is highly heterogeneous, despite CD14 selection removing cells such as lymphocytes which affect osteoclastogenesis and cause significant intra-assay variability [7,13]. Not all of the precursor cell pool responds to the differentiation cytokines, and both murine [14,15] and human [13,16,17] osteoclast preparations contain a mixed population of undifferentiated and mononuclear cells, binuclear pre-osteoclasts and multinucleated osteoclasts at different stages of fusion (3–20+ nuclei). In some murine preparations <20% of cells are osteoclasts [14]. Even greater heterogeneity is introduced when inter-individual donor variation is considered; CD14+ monocytes isolated from multiple donors exhibit an up to 12-fold difference in the number of osteoclasts formed and a >20-fold difference in bone resorption [18,19]. Additionally, osteoclasts cannot be detached from cell culture substrates using standard cell detachment reagents such as trypsin, while mechanical detachment results in loss of viability of large mature osteoclasts [20,21]. These factors combined have necessitated that osteoclast studies to date consider this heterogeneous mono-/bi-/multi-nucleated mixture of cells to be effectively a single population of osteoclasts.

Critically, this assumption makes it difficult to assess the extent to which a disease or drug has an effect on osteoclast formation and/or function. Nevertheless, all work investigating the regulation of mRNA and protein expression during osteoclastogenesis, and in response to drug treatments in both murine [22,23,24,25] and human [17,19,26,27,28] cells, has been performed on such mixed populations. The resulting inclusion of multiple stages of differentiating osteoclasts, undifferentiated monocytes and ‘non-osteoclast’ cells in these analyses introduces significant error and severely hampers the ability to distinguish the transcriptomic and proteomic signatures of different osteoclast stages. It also hinders a more detailed understanding of the importance of this heterogeneity to osteoclastogenesis itself. For example, heterogeneity between fusion partners, with respect to factors such as nuclearity, mobility or expression profile, controls fusion. Additionally, osteoclasts with different levels of multinucleation have distinct properties with respect to gene expression and the sensitivity of their bone-resorbing activity to cytokines within the bone microenvironment [12,29]. The biology of these distinct osteoclast subpopulations is potentially an important issue in the pathology of bone disease.

Some progress has been reported with the purification of murine osteoclasts, which can be detached from cell culture plastic using Accutase and FACS-sorted after DAPI- or Hoechst 33342-staining of the nuclei on the basis of multi-nucleation [14,30]. However, DAPI-staining requires cell fixation, which prevents further functional analysis [14] and, although the Hoescht method could enrich the murine osteoclast population, when applied to human cells there was a substantial reduction in cell viability [30]. Thus, sorting of viable human osteoclasts has so far not been achieved.

This manuscript describes the culture and characterisation of human CD14+ monocyte-derived osteoclasts in 3D collagen gels, and the identification of separate stages of osteoclast development in 3D culture defined by combinations of cell surface markers. This culture method allows the release and live cell sorting of highly enriched viable human osteoclasts from 3D culture with demonstration of full functional resorption capacity, opening the door to precise cell combination experiments as well as in-depth transcriptomic and proteomic analysis of these cells.

## 2. Materials and Methods

### 2.1. Materials and Ethics

The osteoclastogenic cytokines were M-CSF (R&D Systems, Abingdon, UK) and RANKL (Peprotech, London, UK). Unless stated, the other reagents were from Merck Life Science. Use of leucocyte cones for osteoclast differentiation was approved by the London–Fulham Research Ethics Committee (11/H0711/7).

### 2.2. Osteoclast Differentiation

Leucocyte cones were obtained from anonymous donors (NHS Blood and Transplant). CD14+ monocytes were positively selected from the PBMC fraction using magnetic CD14+ microbeads (Miltenyi Biotech, Bisley, UK) according to the manufacturer’s instructions. Monolayer culture: monocytes were seeded at 1 × 10^6^ cells/well in 24-well plates (Corning, 3526) or onto 4 mm diameter dentine discs in 96-well plates (0.25 × 10^6^ cells/well) in α-MEM (without ribonucleosides/deoxyribonucleosides) containing 10% FBS, 2 mM L-glutamine, 50 IU/mL penicillin and 50 µg/mL streptomycin sulphate. Gel-based 3D culture: monocytes were pelleted at 1 × 10^6^ cells per well of a 24-well plate, resuspended in 300 µL of 2 mg/mL collagen type I (Corning) and allowed to polymerize in 24-well plates at 37 °C for 30 min before addition of αMEM. In both culture systems, osteoclastogenesis was induced after overnight incubation in αMEM by the addition of 25 ng/mL M-CSF and 50 ng/mL RANKL. Media and cytokines were replenished every 3–4 days for up to 3 weeks.

### 2.3. Release of Osteoclasts from Monolayer and 3D Culture

Osteoclasts were released from the monolayer culture using Accutase. Media was removed from the monolayer and cells were washed with PBS and then incubated in Accutase at 37 °C for up to 45 min. Osteoclasts were released from collagen gels by digestion with 0.2 mg/mL collagenase type I at 37 °C for 30 min. Cells released from both systems were centrifuged, washed and either resuspended in α-MEM for reseeding for further culture, or in FACS buffer for protein expression analysis or sorting.

### 2.4. Staining for Osteoclast Formation and Activity

TRAP staining: Formalin-fixed osteoclasts were stained for tartrate-resistant acid phosphatase (TRAP) using naphthol AS-BI phosphate as a substrate with reaction of the product with fast violet B. Equal volumes of solution A (10 mg naphthol AS-BI phosphate, 0.5 mL DMSO in 15 mL acetate-tartrate solution [0.2 M acetic acid, 0.2 M sodium acetate, 10 mM sodium tartrate, pH5]) and solution B (20 mg fast violet B salt, 0.5 mL DMSO in 15 mL acetate tartrate solution) were mixed and incubated on fixed cells for 3 h at 37 °C in the dark, prior to washing and air drying. Photographs were obtained on a Nikon Eclipse TE300 microscope with an Axiocam 105 camera (Carl Zeiss AG, Cambridge, UK) and ZEN acquisition software (blue edition; Zeiss). Multinucleated cells with three or more nuclei were considered osteoclasts. CD51/61 staining: immunostaining for osteoclast-specific CD51/61 used an anti-CD51/61 antibody (clone 23C6, 1:400; BioRad, Oxford, UK) and standard DAB immunohistochemistry techniques. Resorption: Sonication was used to remove osteoclasts from dentine discs. Resorption tracks were then visualised by staining with 0.5% toluidine blue in boric acid. Dentines were photographed on an Olympus BX40 microscope with ZEN (blue edition) acquisition software. For quantification, resorption tracks were highlighted in Adobe Photoshop and the relative resorbed area was measured using ImageJ software (Fiji; National Institutes of Health, Bethesda, USA.

### 2.5. Flow Cytometry of Formalin-Fixed Osteoclasts

Cells released from monolayer or 3D culture were centrifuged at 350 g for 5 min, resuspended in FACS staining buffer (PBS, 0.5% BSA, 2 mM EDTA) at 1 × 10^7^ cells/mL and then incubated with Fixable Viability Dye eFluor 780 (VWR International) and FACS antibodies in the dark for 30 min at 4°C. FACS antibodies were as follows: anti-CD51/61-FITC (clone 23C6, 1:20, Biolegend, London, UK), anti-CD9-PE/Cy7 (clone HI9a, 1:20, Biolegend), anti-CD14-PE (clone M5E2, 1:20, Biolegend), anti-CD63-BV711 (clone H5C6, 1:20, Biolegend), anti-RANK-PECy7 (clone 9A725, 1:200, Novusbio, Centennial, CO, USA) and anti-OSCAR (clone REA494, 1:100, Miltenyi Biotech). Cells were washed twice with FACS staining buffer, fixed in 4% formalin for 20 min on ice, washed, and resuspended in FACS staining buffer for analysis. When DAPI staining, fixed cells were incubated with a 1 in 10 volume of DAPI staining solution (10 ug/mL, Miltenyi Biotech) for 15 min at room temperature, then stored at 4 °C in the dark for up to 1 week before analysis. Flow cytometry was performed on a BD Fortessa calibrated with calibration and tracking beads. Forward and side scatter were set to 40 V (FSC) and 210 V (SSC) to enable visualisation of mature osteoclasts within the graphed area, with 10,000 events recorded per sample using FACS Diva software (BD Biosciences). Data were analysed using FlowJo software (FlowJo 10.8.1; BD Biosciences, Wokingham, UK).

### 2.6. Osteoclast Live Cell Sorting

Cells were washed and stained as above. When staining nuclei, two drops of Hoechst 33342 Ready Flow Reagent (Invitrogen, Paisley, UK) were added to 1 × 10^6^ cells in 1 mL media and incubated at 37 °C for 60 min, per the manufacturer’s instructions. Cells were not fixed in formalin, but were resuspended in FACS staining buffer at 0.5 mL/1 × 10^6^ cells and sorted immediately on a Sony SH800 Cell Sorter calibrated with Automatic Set-up Beads, using SH800 software. Sorting was performed with a 100 µm sorting chip at an event rate of 400 and in purity mode, with cells collected in PBS. Data were analysed using FlowJo software. The purity of the enriched osteoclast population was checked by flow cytometry of a sample of the sorted cells.

### 2.7. Statistics

Graphical results are presented with the number of experimental repeats indicated by the number of data points. Error bars indicate standard deviation. Data were analysed using GraphPad Prism (v9.4.1; GraphPad Software, La Jolla, CA, USA). D’Agistono–Pearson or Shapiro–Wilk were used to test for normality, depending on the sample size. Statistical analysis comprised one-way or two-way ANOVA using Dunnett’s or Tukey’s multiple comparison as a post hoc test. For experiments with only two conditions, a T-test was applied. Results were considered significant at *p* < 0.05.

## 3. Results

### 3.1. Cellular Comparison of Monolayer and 3D-Generated Human Osteoclasts

**Figure 1:**
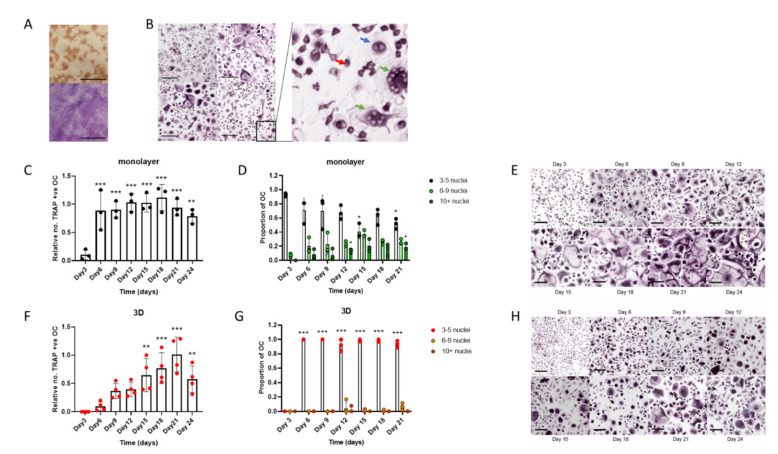
Human osteoclasts differentiated in 3D form a more homogeneous cell population than monolayer cells. (**A**–**E**) *Monolayer osteoclasts*: (**A**) Representative images of staining for CD51/61 (top) and visualisation of resorption tracks (bottom, scale bars = 200 µm) and (**B**) TRAP staining of osteoclasts from 4 independent donors (scale bar = 200 µm) on day 9 of differentiation showing heterogeneity of the populations; (insert) monocytes (red arrow), binuclear pre-osteoclasts (blue arrow) and mature osteoclasts (green arrow). (**C**) Rate of formation of monolayer osteoclasts (TRAP-positive cells with ≥3 nuclei) and (**D**) the size distribution of these osteoclasts; small (3–5 nuclei), medium (6–9 nuclei) or large (>10 nuclei). (**E**) Representative TRAP-stained images of the time course of osteoclast differentiation in monolayer culture (scale bar = 100 µm). (**F**–**H**) *3D-generated osteoclasts:* Cells were fixed for analysis 4 h after reseeding onto cell culture plates (**F**) Rate of formation of 3D-generated osteoclasts (TRAP-positive cells with ≥3 nuclei) and (**G**) the size distribution of these osteoclasts; small (3–5 nuclei), medium (6–9 nuclei) or large (>10 nuclei). (**H**) Representative TRAP-stained images of the time course of osteoclast differentiation in 3D (scale bar = 100 µm). *, *p* < 0.05; **, *p* < 0.01; ***, *p* < 0.001.

Standard differentiation of human osteoclasts from CD14+ monocyte precursors in monolayer culture (on cell culture plastic, glass coverslips or dentine discs), using M-CSF and RANKL, produced mature CD51/61-positive bone-resorbing osteoclasts by day 9 of differentiation (Figure 1A). The osteoclast preparations were highly heterogeneous, containing a mixture of TRAP-positive mononuclear cells, binuclear pre-osteoclasts and multinucleated osteoclasts at different stages of fusion (3–20+ nuclei) (Figure 1B). When the differentiation period was extended to 24 days, there was no increase in total osteoclast number (Figure 1C); however, fusion continued throughout this period, and the proportion of medium-sized (6–9 nuclei) and large (≥10 nuclei) osteoclasts increased (Figure 1D,E).

We have previously described the differentiation of human osteoclasts in 3D collagen gels [16,31]. To characterise these populations, cells were released from the gel with collagenase and reseeded onto cell culture plates. Cells were fixed 4 h after reseeding, a period that was found to allow cell adhesion but to be too early for further cell fusion to occur. In comparison with monolayer cells, 3D-generated osteoclasts fused more slowly, with the maximum number of osteoclasts being achieved on day 21 of differentiation (Figure 1F). TRAP-positive mononuclear cells and binuclear pre-osteoclasts were also present, as with monolayer culture; however, most osteoclasts remained small (3–5 nuclei) resulting in greater homogeneity of the osteoclast population than was observed with cells differentiated in monolayer (Figure 1G,H).

### 3.2. Osteoclasts Generated in 3D Retain Viability when Re-Seeded Following Release from Culture

**Figure 2:**
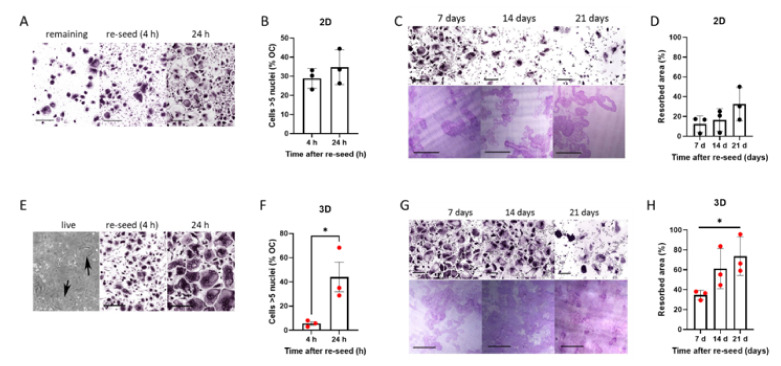
Rapid fusion and prolonged resorption by 3D osteoclasts reseeded onto solid substrates. (**A**–**D**) *Monolayer osteoclasts*: (**A**) TRAP-staining of monolayer osteoclasts after treatment with Accutase either (left) remaining adherent to the original cell culture well or (middle, right) reseeded into clean cell culture wells for 4 h or 24 h (scale bar = 200 µm), quantified (**B**) as the percentage of osteoclasts with 5 or more nuclei. (**C**) TRAP-staining (top) and toluidine blue-stained resorption pits on dentine (bottom) of monolayer osteoclasts reseeded on day 15 of differentiation, shown 7–21 days post-reseed. Scale bars = 200 µm. (**D**) Quantification of area of bone resorbed. (**E**–**H**) *3D-generated osteoclasts*: (**E**) Phase contrast image of 3D-generated osteoclasts in a collagen gel (left panel, arrows indicate osteoclasts), TRAP staining of cells 4 h after collagenase release and reseeding (middle panel, scale bar = 200 µm) and 24 h after reseeding (right panel, scale bar = 200 µm), quantified (**F**) as the percentage of osteoclasts with 5 or more nuclei. (**G**) TRAP-staining (top) and toluidine blue-stained resorption pits on dentine (bottom) of 3D-generated osteoclasts reseeded on day 15 of differentiation, shown 7–21 days post-reseed. Scale bars = 200 µm. (**H**) Quantification of area of bone resorbed. *, *p* < 0.05.

Accutase can be used to viably release murine osteoclasts from monolayer culture [14,30]. Accutase treatment released a variable proportion of human osteoclasts from monolayer culture on cell culture plastic; substantial numbers of osteoclasts remained adherent even after 45 min incubation (Figure 2A). The reseeded monolayer osteoclasts exhibited bone resorption activity for at least 7 days, despite visibly reduced viability from 14–21 days post re-seed (Figure 2B–D). Despite the restraint on fusion in 3D culture, 3D-generated osteoclasts released with collagenase fused rapidly once reseeded onto a solid substrate, showing a 7.8-fold increase in the proportion of osteoclasts with >5 nuclei between 4 and 24 h (Figure 2E,F). They exhibited a strong capacity for bone resorption, remaining active for at least 3 weeks (Figure 2G,H) and with visibly reduced viability only 21 days post re-seed. It should be noted that when monolayer and 3D-generated cells were released from culture and immediately fixed for flow cytometry, viability was similar and unaffected by the duration of differentiation pre-release (Figure S1). This suggests that the reduced lifespan of the monolayer osteoclasts that do re-adhere is not due to immediate loss of viability following exposure to Accutase, but that previously adherent cells undergo cell death more rapidly following reattachment.

### 3.3. FACS Analysis of Cell Surface Marker Proteins Distinguishes Osteoclast Subpopulations Following Differentiation in 3D

**Figure 3:**
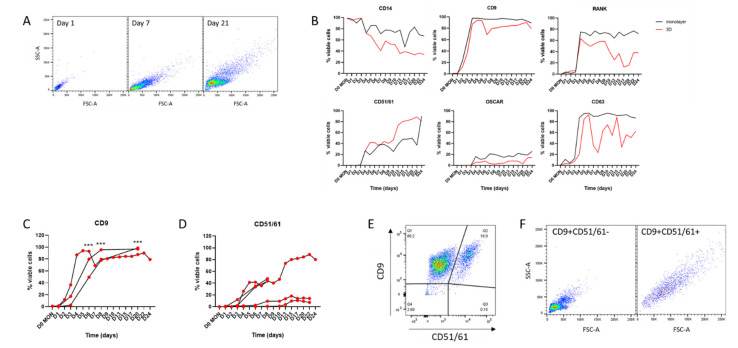
Cell surface markers in monolayer and 3D osteoclasts. (**A**) FACS scatter graphs showing increasing cell size and complexity during differentiation from CD14+ monocytes to osteoclasts in 3D culture. (**B**) Representative data from cells from one donor showing variation in osteoclast surface markers over 24 days of differentiation. Black = monolayer cells, red = 3D-generated cells. (**C**,**D**) Comparison of expression of (**C**) CD9 (n = 3) and (**D**) CD51/61 (n = 5) during 3D differentiation of osteoclasts from different monocyte donors. (**E**) Double staining of 3D-generated osteoclasts on day 15 of differentiation for CD9 and CD51/61 distinguishes two osteoclast subpopulations. (**F**) Scatter graph of CD9+CD51/61−cells showing small early/pre-osteoclasts (left panel) with CD9+CD51/61+ cells being larger mature osteoclasts (right panel). ***, *p* < 0.001.

We first sought to compare the expression of osteoclast cell surface marker proteins in formalin-fixed monolayer and 3D-generated cells by flow cytometry. The observed scatter pattern was similar to that reported for murine osteoclasts [30], with scatter increasing on both axes as monocytes fused and differentiated into osteoclasts (Figure 3A). Expression of osteoclast surface marker proteins was compared in 3D versus monolayer culture using monocytes from the same donor that were differentiated into osteoclasts and stained and fixed daily for FACS. There was generally good concordance in expression of monocyte markers (CD14), proteins related to early osteoclast differentiation and fusion (RANK, CD9) and markers of more mature osteoclasts (CD51/61, OSCAR, CD63) (Figure 3B), suggesting that cells in 3D collagen gels were differentiating at approximately the same rate as those in standard monolayer culture with respect to osteoclast marker proteins, despite reduced fusion.

Osteoclasts expressing specific combinations of cell surface markers represent distinct subpopulations of human osteoclasts at different stages of differentiation. For example, CD9 is an early regulator of cell fusion during osteoclastogenesis [32]. Analysis of CD9 expression in 3D-generated osteoclast populations from multiple independent donors confirmed that it is reproducibly upregulated, so that >50% of cells express CD9 by day 6 of differentiation and >80% of cells are CD9+ from day 8 (Figure 3C). Analysis of CD51/61 expression (a marker of mature osteoclasts that is not expressed in either mononuclear cells or pre-osteoclasts [33]) showed that it is consistently upregulated by 2 weeks of differentiation in 3D-generated osteoclasts (Figure 3D). However, the proportion of CD51/61+ cells in these populations was highly variable (9.78–80.2% CD51/61-positive at 15 days), highlighting the inherent heterogeneity both within and between osteoclast populations, especially regarding late markers of differentiation. By using these cell surface markers it is possible to distinguish three subpopulations of cells in a population of differentiating osteoclasts: fully mature CD9+CD51/61+ osteoclasts, undifferentiated CD9−CD51/61− monocytes and CD9+CD51/61− cells that can be considered as pre-/early osteoclasts (Figure 3E,F). Given the heterogeneity between donors (Figure 3D), the ability to sort selected subpopulations of viable human osteoclasts would greatly increase the reproducibility of experiments using multiple donors.

### 3.4. Live Cell Sorting of Viable, Mature Human Osteoclasts from 3D Culture

**Figure 4:**
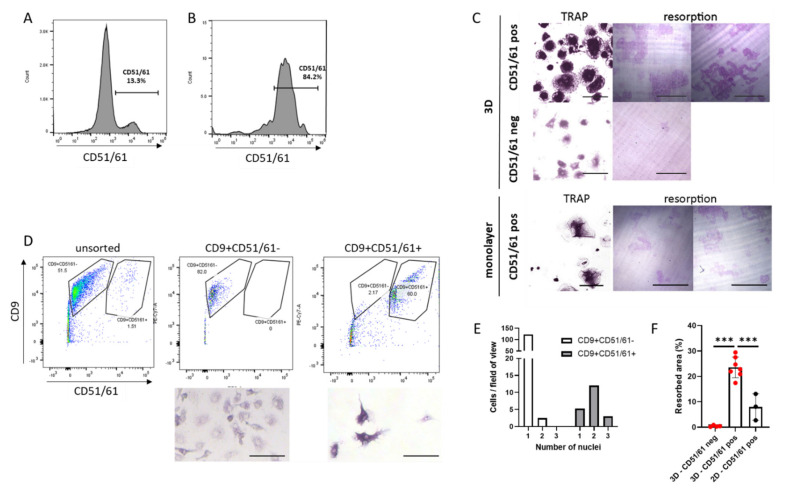
Live cell sorting of primary human osteoclasts. (**A**) Flow cytometry of live 3D-generated human osteoclasts stained for CD51/61. (**B**) Following cell sorting, the population of CD51/61+ cells increased from 13.3% to 84.2%. (**C**) Cells re-seeded into monolayer culture after live cell sorting and analysed for the presence of osteoclasts (TRAP stain, 24 h post re-seed, scale bar = 100 µm) and resorption activity (resorption tracks on dentine, 7 days post re-seed, scale bar = 200 µm). Top panel: CD51/61-positive cells from 3D culture. Middle panel: CD51/61-negative cells from 3D culture. Bottom panel: CD51/61-positive cells from monolayer culture. (**D**) Flow cytometry of live 3D-generated human osteoclasts stained for CD9 and CD51/61. CD9+CD51/61- and CD9+CD51/61+ cells were re-seeded into monolayer culture after live cell sorting and analysed for the presence of osteoclasts 24 h post re-seed (TRAP stain, scale bar = 100 µm), quantified (**E**) as the number of nuclei per cell in these selected populations. (**F**) Area of bone resorbed by monolayer and 3D-generated osteoclasts selected for CD51/61. Black = monolayer cells, red = 3D-generated cells. ***, *p* < 0.001.

Although live cell sorting is a standard procedure for many cell types, it has not been achieved with human osteoclasts. CD51/61 was selected as the cell surface marker to optimise osteoclast sorting, as the proportion of CD51/61+ cells in the population of 3D-generated osteoclasts was highly variable and frequently low (Figure 3D). After 2 weeks of differentiation, 3D osteoclasts were released from the gel with collagenase, stained for CD51/61 and sorted by flow cytometry, resulting in a highly enriched population of mature osteoclasts. As an example, a 3D osteoclast population containing only 13.3% mature CD51/61+ osteoclasts (Figure 4A) was enriched to 84.2% CD51/61+ cells (Figure 4B). Crucially, sorted osteoclasts retained bone resorption activity when re-plated onto dentine discs (Figure 4C), verifying the ability of osteoclasts generated in 3D to be sorted into selected populations of viable and active human osteoclasts. As an extension of this methodology, both CD9+CD51/61− and CD9+CD51/61+ cells were selected from a younger population of 3D-generated osteoclasts on day 9 of differentiation. Intermediate CD9+CD51/61− immature osteoclasts were enriched from 51.5% to 82.0% and contained cells with only one nucleus (Figure 4D,E). Mature CD9+CD51/61+ cells comprising only 1.51% of the population were highly enriched to 60.0% of CD9+CD51/61+ osteoclasts, the majority of which were osteoclasts with two or three nuclei (Figure 4D,E).

Considering the success of this methodology, we investigated whether osteoclasts differentiated in monolayer culture and lifted with Accutase could also be viably sorted using cell surface markers. CD14+ monocytes from the same donor were differentiated in monolayer and 3D culture. An equal number of cells released from each culture system were sorted to obtain CD51/61+ cells, which were seeded onto the same area of cell culture plastic or dentine. Fewer CD51/61+ osteoclasts were obtained from the monolayer population and less bone was resorbed compared with 3D-generated osteoclasts (Figure 4C,F). This comparison emphasises the importance of using smaller 3D-generated osteoclasts for maintaining viability and resorption capacity following live cell-sorting.

## 4. Discussion

The development of novel osteoclast-targeting strategies is currently hindered by the technical inability to obtain pure and distinct populations of viable human osteoclasts for research. This study shows for the first time that human osteoclasts can be sorted and selected using cell surface markers, and that they can be viably re-seeded retaining full bone-resorption capacity. The data also show that, while this is possible with monolayer osteoclasts to a limited extent, osteoclasts differentiated in 3D culture can be viably sorted in much greater numbers and with extended subsequent viability and function, offering a valuable new approach to dissecting osteoclast biology.

With monolayer osteoclasts, the low numbers of cells obtained and the loss of viability following cell sorting is likely due to a combination of factors including: (i) retained adhesion to cell culture plastic of a large proportion of osteoclasts following incubation with Accutase, (ii) potential further loss of large osteoclasts during cell sorting and (iii) reduced viability of monolayer osteoclasts after re-plating. It is possible that use of Accutase versus collagenase could have affected the viability of monolayer osteoclasts after sorting, despite having shown that immediate viability post-release is not different. However, as neither type of osteoclast can be detached using the reagent for the other, this comparison cannot be performed directly. However, our sorting of monolayer osteoclasts was still an improvement on that of Madel et al., who were unable to sort viable human osteoclasts on the basis of multinucleation by staining with Hoechst 33342, despite the success of this technique with murine cells [30]. Our attempts to sort human osteoclasts using Hoescht 33342 were also unsuccessful; whether using monolayer- or 3D-generated cells, all subsequent cultures failed (data not shown), suggesting that Hoescht 33342 is toxic to human osteoclasts.

Human osteoclasts cultured on hydrophobic dishes remain viable and produce resorption pits when re-seeded onto dentine discs, although the reseeding process can require application of a detachment reagent such as Accutase [34,35]. We therefore hypothesised that reduced adhesion and/or reduced polarisation of differentiating osteoclasts could aid retention of cell viability and resorption activity upon reseeding; this led us to investigate the advantages of differentiating osteoclasts in 3D collagen gels. As hypothesised, osteoclasts released from 3D culture remained viable and active for 2–3 weeks after reseeding onto dentine discs.

Osteoclasts differentiated in 3D collagen gels remained small (3–5 nuclei) even after three weeks of differentiation. This did not apparently alter the rate of production of differentiation-related proteins in a direct comparison of monolayer and 3D-generated cells. It is possible that, despite monolayer osteoclasts being morphologically more mature, similar expression of differentiation markers was seen in monolayer versus 3D culture because Accutase preferentially released a higher proportion of undifferentiated and immature osteoclasts from cell culture plastic. However, this limitation on the comparison cannot be addressed until improved methods of detaching osteoclasts from monolayer culture are developed.

The size restriction affecting 3D-generated osteoclasts produced a more homogeneous osteoclast population in terms of multi-nucleation than for monolayer cells. It is of interest that the fusion process requires heterogeneity, including differences in the differentiation state and number of nuclei of fusion partners, as well as in the mobility of the fusion precursors [12,36]. The new potential to distinguish and select multiple populations of osteoclasts at different stages of differentiation from this 3D population opens the door to specific cell-based studies into both the mechanism(s) of fusion between different combinations of fusion precursors, and the properties of osteoclasts at different stages of differentiation. This offers the opportunity to build a more detailed picture of osteoclastogenesis than has previously been possible and, crucially, would enable the first complete experimental separation of the effects of a treatment or co-culture on osteoclast formation versus mature osteoclast function.

It is interesting to speculate whether monolayer or 3D-generated osteoclasts are more physiologically relevant. We have previously used RNAseq to show distinct clustering of the gene expression profile of monolayer osteoclasts differentiated on either cell culture plastic, acellular cartilage or dentine [19]. It could be presumed that 3D-generated osteoclasts would show a similarly distinct profile. However, osteoclasts in vivo are located adjacent to and actively resorbing bone and cartilage, as well as being found distant from either substrate in conditions such as primary bone cancer and rheumatoid arthritis. These osteoclasts in vivo are likely to also have distinct expression profiles. The main advantage of the 3D-generated osteoclasts is the ability to sort these cells without loss of viability. As we have also shown a more limited capacity to sort monolayer osteoclasts, the combination of differentiation methods and osteoclast sorting expands the repertoire of possible experiments that can be used to compare with in vivo osteoclast profiles, tissue and clinical outcomes. While the current methodology would require some optimisation to improve the final purity of the desired osteoclast subpopulation(s), especially where the starting population is small, it should be noted that 40-fold enrichment of a 1.51% subpopulation has already been achieved, and that the sorted purity of the final selected subpopulations generally exceeds 80%.

Osteoclasts generated in 3D might also be used to understand the processes that modify osteoclast formation or bone resorption activity in pathologies ranging from osteopetrosis to osteoporosis, and from chronic inflammation to cancer, using sorted subpopulations for in-depth sequencing and proteomics studies, as well as for functional assays. While single-cell RNA sequencing has been applied to osteoclast precursor cells, identifying the specific macrophage subpopulation that differentiates into pathological mature osteoclasts in inflammatory arthritis [37], our methodology now offers avenues by which to explore in detail the nature of the different populations of immature and mature osteoclasts that are subsequently formed. For example, osteoclasts are a specific subtype of macrophage polykaryon, currently distinguished only by osteoclasts’ expression of the calcitonin receptor and ability to resorb bone [38]. Separation of the two populations would allow detailed analysis of possible additional distinguishing markers.

Osteoclast cell sorting also offers opportunities to study the interaction of specific osteoclast subsets within the tumour microenvironment, including expanding recent research into their role in the modulation of T cell activation towards either immune suppression or inflammation [39,40]. The ability to distinguish and select mature or differentiating osteoclast populations will also allow improved understanding of effects of the microenvironment. The role of the mechanical environment and stiffness versus softness in osteoclastogenesis would be of interest and tractable to study. Additional examples include whether components of human calcified atherosclerotic plaques, such as high levels of inorganic phosphate [41,42], inhibit the resorption activity of mature cells or just affect the process of osteoclast differentiation [43]. Mature osteoclasts actively reduce the mineral load of pre-calcified aortic elastin in vitro [44], suggesting that direct introduction of mature osteoclasts might one day lead to autologous cell therapies to resorb calcified vascular lesions.

## 5. Conclusions

The combination of differentiating primary human osteoclasts in 3D with live cell-sorting using cell surface markers allows the release from culture and significant enrichment of viable human osteoclasts, which retain full resorption capacity. Differentiation in 3D collagen gels offers new opportunities to manipulate and purify human osteoclasts, opening the door to in-depth transcriptomic and proteomic analysis of selected subpopulations of these cells that will greatly increase our ability to study human osteoclast molecular mechanisms relevant to development, aging and disease.

## Data Availability

All data generated or analysed during this study are included in this published article.

## Supplementary Materials

The following supporting information can be downloaded at: https://www.mdpi.com/article/10.3390/cells11243973/s1. Figure S1. Cell viability immediately after release from monolayer or 3D culture.

## References

[1] McDonald M.M., Kim A.S., Mulholland B.S., Rauner M. (2021). New Insights into Osteoclast Biology. JBMR Plus.

[2] Gambari L., Grassi F., Roseti L., Grigolo B., Desando G. (2020). Learning from Monocyte-Macrophage Fusion and Multinucleation: Potential Therapeutic Targets for Osteoporosis and Rheumatoid Arthritis. Int. J. Mol. Sci..

[3] Dewulf J., Vangestel C., Verhoeven Y., van Dam P., Elvas F., Van den Wyngaert T., Clezardin P. (2019). Bone metastases in the era of targeted treatments: Insights from molecular biology. Q. J. Nucl. Med. Mol. Imaging.

[4] Marino S., Logan J.G., Mellis D., Capulli M. (2014). Generation and culture of osteoclasts. BoneKEy Rep..

[5] Knowles H.J., Cleton-Jansen A.M., Korsching E., Athanasou N.A. (2010). Hypoxia-inducible factor regulates osteoclast-mediated bone resorption: Role of angiopoietin-like 4. FASEB J..

[6] Massey H.M., Flanagan A.M. (1999). Human osteoclasts derive from CD14-positive monocytes. Br. J. Haematol..

[7] Nicholson G.C., Malakellis M., Collier F.M., Cameron P.U., Holloway W.R., Gough T.J., Gregorio-King C., Kirkland M.A., Myers D.E. (2000). Induction of osteoclasts from CD14-positive human peripheral blood mononuclear cells by receptor activator of nuclear factor kappaB ligand (RANKL). Clin. Sci..

[8] Fujikawa Y., Quinn J.M., Sabokbar A., McGee J.O., Athanasou N.A. (1996). The human osteoclast precursor circulates in the monocyte fraction. Endocrinology.

[9] Quinn J.M., Elliott J., Gillespie M.T., Martin T.J. (1998). A combination of osteoclast differentiation factor and macrophage-colony stimulating factor is sufficient for both human and mouse osteoclast formation in vitro. Endocrinology.

[10] Yasuda H., Shima N., Nakagawa N., Yamaguchi K., Kinosaki M., Mochizuki S., Tomoyasu A., Yano K., Goto M., Murakami A. (1998). Osteoclast differentiation factor is a ligand for osteoprotegerin/osteoclastogenesis-inhibitory factor and is identical to TRANCE/RANKL. Proc. Natl. Acad. Sci. USA.

[11] Kurotaki D., Yoshida H., Tamura T. (2020). Epigenetic and transcriptional regulation of osteoclast differentiation. Bone.

[12] Takito J., Nakamura M. (2020). Heterogeneity and Actin Cytoskeleton in Osteoclast and Macrophage Multinucleation. Int. J. Mol. Sci..

[13] Henriksen K., Karsdal M.A., Taylor A., Tosh D., Coxon F.P. (2012). Generation of human osteoclasts from peripheral blood. Methods Mol. Biol..

[14] Ibanez L., Abou-Ezzi G., Ciucci T., Amiot V., Belaid N., Obino D., Mansour A., Rouleau M., Wakkach A., Blin-Wakkach C. (2016). Inflammatory Osteoclasts Prime TNFalpha-Producing CD4(+) T Cells and Express CX3 CR1. J. Bone Miner. Res..

[15] Hajjawi M.O., Patel J.J., Corcelli M., Arnett T.R., Orriss I.R. (2016). Lack of effect of adenosine on the function of rodent osteoblasts and osteoclasts in vitro. Purinergic Signal..

[16] Knowles H.J. (2020). Distinct roles for the hypoxia-inducible transcription factors HIF-1alpha and HIF-2alpha in human osteoclast formation and function. Sci. Rep..

[17] Sorensen M.G., Henriksen K., Schaller S., Henriksen D.B., Nielsen F.C., Dziegiel M.H., Karsdal M.A. (2007). Characterization of osteoclasts derived from CD14+ monocytes isolated from peripheral blood. J. Bone Miner. Metab..

[18] Moller A.M.J., Delaisse J.M., Olesen J.B., Canto L.M., Rogatto S.R., Madsen J.S., Soe K. (2020). Fusion Potential of Human Osteoclasts In Vitro Reflects Age, Menopause, and In Vivo Bone Resorption Levels of Their Donors-A Possible Involvement of DC-STAMP. Int. J. Mol. Sci.

[19] Larrouture Q.C., Cribbs A.P., Rao S.R., Philpott M., Snelling S.J., Knowles H.J. (2021). Loss of mutual protection between human osteoclasts and chondrocytes in damaged joints initiates osteoclast-mediated cartilage degradation by MMPs. Sci. Rep..

[20] Chambers T.J. (1979). Phagocytosis and trypsin-resistant glass adhesion by osteoclasts in culture. J. Pathol..

[21] Fuller K., Kirstein B., Chambers T.J. (2006). Murine osteoclast formation and function: Differential regulation by humoral agents. Endocrinology.

[22] Czupalla C., Mansukoski H., Pursche T., Krause E., Hoflack B. (2005). Comparative study of protein and mRNA expression during osteoclastogenesis. Proteomics.

[23] Chen Y., Zhang L., Li Z., Wu Z., Lin X., Li N., Shen R., Wei G., Yu N., Gong F. (2022). Mogrol Attenuates Osteoclast Formation and Bone Resorption by Inhibiting the TRAF6/MAPK/NF-kappaB Signaling Pathway In vitro and Protects Against Osteoporosis in Postmenopausal Mice. Front. Pharmacol..

[24] Estell E.G., Le P.T., Vegting Y., Kim H., Wrann C., Bouxsein M.L., Nagano K., Baron R., Spiegelman B.M., Rosen C.J. (2020). Irisin directly stimulates osteoclastogenesis and bone resorption in vitro and in vivo. eLife.

[25] Guerit D., Marie P., Morel A., Maurin J., Verollet C., Raynaud-Messina B., Urbach S., Blangy A. (2020). Primary myeloid cell proteomics and transcriptomics: Importance of beta-tubulin isotypes for osteoclast function. J. Cell Sci..

[26] Liu W., Li Z., Cai Z., Xie Z., Li J., Li M., Cen S., Tang S., Zheng G., Ye G. (2020). LncRNA-mRNA expression profiles and functional networks in osteoclast differentiation. J. Cell Mol. Med..

[27] Rimondi E., Zweyer M., Ricci E., Fadda R., Secchiero P. (2007). Receptor activator of nuclear factor kappa B ligand (RANKL) modulates the expression of genes involved in apoptosis and cell cycle in human osteoclasts. Anat. Rec..

[28] Aitken C.J., Hodge J.M., Nishinaka Y., Vaughan T., Yodoi J., Day C.J., Morrison N.A., Nicholson G.C. (2004). Regulation of human osteoclast differentiation by thioredoxin binding protein-2 and redox-sensitive signaling. J. Bone Miner. Res..

[29] Soe K. (2020). Osteoclast Fusion: Physiological Regulation of Multinucleation through Heterogeneity-Potential Implications for Drug Sensitivity. Int. J. Mol. Sci..

[30] Madel M.B., Ibanez L., Rouleau M., Wakkach A., Blin-Wakkach C. (2018). A Novel Reliable and Efficient Procedure for Purification of Mature Osteoclasts Allowing Functional Assays in Mouse Cells. Front. Immunol..

[31] Hulley P.A., Papadimitriou-Olivgeri I., Knowles H.J. (2020). Osteoblast-Osteoclast Coculture Amplifies Inhibitory Effects of FG-4592 on Human Osteoclastogenesis and Reduces Bone Resorption. JBMR Plus.

[32] Ishii M., Iwai K., Koike M., Ohshima S., Kudo-Tanaka E., Ishii T., Mima T., Katada Y., Miyatake K., Uchiyama Y. (2006). RANKL-induced expression of tetraspanin CD9 in lipid raft membrane microdomain is essential for cell fusion during osteoclastogenesis. J. Bone Miner. Res..

[33] Horton M.A., Lewis D., McNulty K., Pringle J.A., Chambers T.J. (1985). Monoclonal antibodies to osteoclastomas (giant cell bone tumors): Definition of osteoclast-specific cellular antigens. Cancer Res..

[34] Hemingway F., Cheng X., Knowles H.J., Estrada F.M., Gordon S., Athanasou N.A. (2011). In vitro generation of mature human osteoclasts. Calcif. Tissue Int..

[35] Heinemann C., Adam J., Kruppke B., Hintze V., Wiesmann H.P., Hanke T. (2021). How to Get Them off?-Assessment of Innovative Techniques for Generation and Detachment of Mature Osteoclasts for Biomaterial Resorption Studies. Int. J. Mol. Sci..

[36] Moller A.M., Delaisse J.M., Soe K. (2017). Osteoclast Fusion: Time-Lapse Reveals Involvement of CD47 and Syncytin-1 at Different Stages of Nuclearity. J. Cell Physiol..

[37] Hasegawa T., Ishii M. (2022). Pathological Osteoclasts and Precursor Macrophages in Inflammatory Arthritis. Front. Immunol..

[38] Quinn J.M., Morfis M., Lam M.H., Elliott J., Kartsogiannis V., Williams E.D., Gillespie M.T., Martin T.J., Sexton P.M. (1999). Calcitonin receptor antibodies in the identification of osteoclasts. Bone.

[39] Madel M.B., Ibanez L., Wakkach A., de Vries T.J., Teti A., Apparailly F., Blin-Wakkach C. (2019). Immune Function and Diversity of Osteoclasts in Normal and Pathological Conditions. Front. Immunol..

[40] Madel M.B., Ibanez L., Ciucci T., Halper J., Rouleau M., Boutin A., Hue C., Duroux-Richard I., Apparailly F., Garchon H.J. (2020). Dissecting the phenotypic and functional heterogeneity of mouse inflammatory osteoclasts by the expression of Cx3cr1. eLife.

[41] M’Baya-Moutoula E., Louvet L., Metzinger-Le Meuth V., Massy Z.A., Metzinger L. (2015). High inorganic phosphate concentration inhibits osteoclastogenesis by modulating miR-223. Biochim. Biophys. Acta.

[42] Li J., Xing G., Zhang L., Shang J., Li Y., Li C., Tian F., Yang X. (2017). Satb1 promotes osteoclastogenesis by recruiting CBP to upregulate miR-223 expression in chronic kidney disease-mineral and bone disorder. Pharmazie.

[43] Henaut L., Candellier A., Boudot C., Grissi M., Mentaverri R., Choukroun G., Brazier M., Kamel S., Massy Z.A. (2019). New Insights into the Roles of Monocytes/Macrophages in Cardiovascular Calcification Associated with Chronic Kidney Disease. Toxins.

[44] Simpson C.L., Lindley S., Eisenberg C., Basalyga D.M., Starcher B.C., Simionescu D.T., Vyavahare N.R. (2007). Toward cell therapy for vascular calcification: Osteoclast-mediated demineralization of calcified elastin. Cardiovasc. Pathol..

