# Supplementary material for: A New Method to Sort Differentiating Osteoclasts into Defined Homogeneous Subgroups
Source: Cells. 2022 Dec 8;11(24):3973. doi: 10.3390/cells11243973 (PMC9777285; doi:10.3390/cells11243973)
Supplement: Supplementary file 1 [file cells-11-03973-s001.zip › cells-2051878-supplementary.pdf]

Supplementary Figure S1

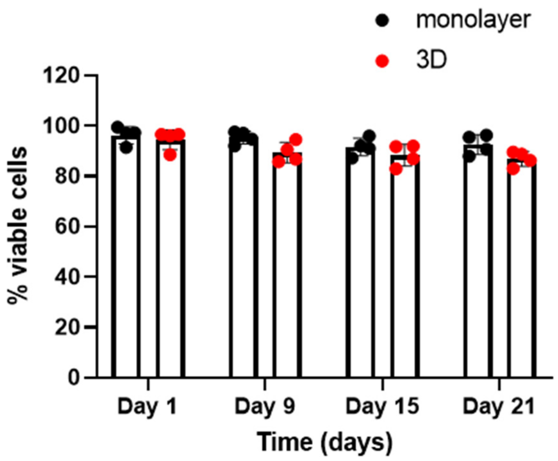


**Figure S1.** Cell viability immediately after release from monolayer or 3D culture. Days indicate duration of differentiation pre-release. Viability is expressed as a percentage of total cell number, assessed by flow cytometry using the eF780 viability dye. $p < 0.05$.
